# Role of the putative *sit1* gene in normal germination of spores and virulence of the *Mucor lusitanicus*

**DOI:** 10.15698/mic2025.08.856

**Published:** 2025-08-12

**Authors:** Bernadett Vágó, Kitti Bauer, Naomi Varghese, Sándor Kiss-Vetráb, Sándor Kocsubé, Mónika Varga, András Szekeres, Csaba Vágvölgyi, Tamás Papp, Gábor Nagy

**Affiliations:** 1 Department of Biotechnology and Microbiology, University of Szeged, Szeged, Közép fasor 52, Hungary.; 2 HUN-REN-SZTE Fungal Pathomechanisms Research Group, University of Szeged, Szeged, Hungary, Szeged, Közép fasor 52, Hungary.; 3 University of Szeged, Centre of Excellence for Interdisciplinary Research, Development and Innovation (SZTE IKIKK), Fungal Pathomechanisms Research Group, Szeged, Hungary, Szeged, Közép fasor 52, Hungary.; a These authors contributed equally to this work.

**Keywords:** mucormycosis, siderophore, Sit1/ARN3, deferoxamine, iron acquisition

## Abstract

Mucormycosis is a life-threatening infection caused by certain members of the fungal order Mucorales, with increased incidence in recent years. Individuals with untreated diabetes mellitus, and patients treated with deferoxamine are particularly susceptible to this infection. Elevated free iron concentrations in serum contribute to the development of mucormycosis. Pathogenic fungi have evolved multiple mechanisms to acquire and utilize free iron or extract it from the various iron-binding molecules within the host. The utilization of hydroxamate siderophores as xenosiderophores may contribute to the development of mucormycosis. The genome of *Mucor lusitanicus* encodes one Sit1 siderophore transporter. In this study, the role of the *sit1* gene was characterized by generating knockout mutants using CRISPR-Cas9. Relative transcript level of the *sit1* gene significantly increased in the presence of deferoxamine- and deferasirox-iron complexes. Lack of *sit1* resulted in altered germination of spores and growth ability, and decreased virulence. Furthermore, absence of the gene caused elevated transcript levels of a ferric reductase (FRE), a low-affinity iron permease (FET4) and a copper dependent iron oxidase (FET3). Our result suggests that expressions of the genes involved in iron uptake affect each other. The lack of Sit1 resulted in an increased transcript level of the *FRE3* gene, which may be able to reduce iron from the siderophore-iron complex. The reduced and liberated iron may be then taken up by activated FET4a. This study highlights the significance of understanding the iron acquisition mechanisms of pathogenic fungi to develop effective treatments for fungal infections.

## Abbreviations

FRAP - ferric reducing assay,

*MFS - major facilitator superfamily.*

## INTRODUCTION

Mucormycosis is a life-threatening opportunistic infection caused by certain members of the fungal order Mucorales 1,2,3,4,5,6. In clinic, *Rhizopus* spp. are the most prevalent agents of mucormycosis followed by *Mucor* and *Lichtheimia* species 3,4,7. These infections are associated with a rapid progression and high mortality rate, which can reach from 30 to nearly 100% depending on the underlying condition of the patient, the clinical manifestation and the treatment strategy 5,8. The main risk factors for the infection are the immunocompromised state due to hematological malignancies 5,8,9, immunosuppressive treatment 9,10, corticosteroid treatment 11, skin burn and trauma 8, uncontrolled diabetes 7,8,12,13,14, elevated serum iron levels 15, and iron-chelating treatment (deferoxamine) 16. Recently, an increasing number of cases has been reported with uncontrolled diabetes undergoing COVID-19 disease worldwide 17,18,19,20,21. As a result, WHO declared Mucorales fungi as high priority group pathogens in 2022 and urges the improvement of research, development and public health actions 22.

Iron is an essential nutrient for both the host and the pathogen. Among others, it is used as the co-factors of several enzymes, such as those catalyzing redox processes 23. Although iron is present in large concentrations in nature, in an aerobic environment, it generally is in an insoluble form, which is difficult to utilize by microorganisms. Therefore, microbes developed basically three strategies to increase the solubility of iron and acquire it, i.e., acidification of the environment, reduction of ferric iron to the more soluble ferrous form and secretion and uptake of soluble iron-chelating molecules 24. In mammalian hosts, serum contains a very low concentration of free iron (10^-18 ^M) due to iron binding proteins, such as transferrin and lactoferrin 15,25. Pathogenic fungi use various strategies to utilize the free iron or liberate it from the different iron binding molecules within the host.

**Figure 1:**
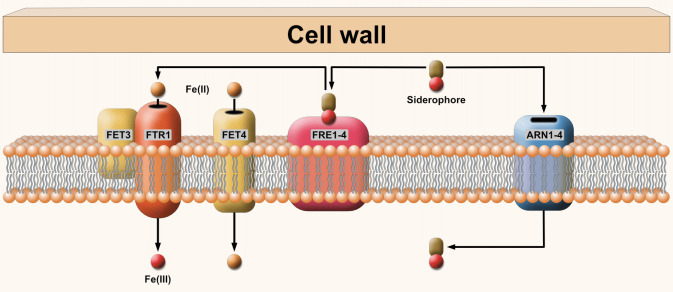
FIGURE 1: Transporters involved in iron uptake in *S. cerevisiae*. Arn1: ferrichrome transporter; Arn2: TAFC transporter; Arn3: hydroxamate siderophore transporter; Arn4: enterobactin transporter; FRE1 and FRE2: mettaloreductases; FRE3: sideorophre reductase; FRE4: ferric reductase; FET4: low affinity (Fe(II)) iron transporter; FTR1: permease; FET3: multicopper oxidase 47. Brown ellipsis indicates siderophore, red circle is Fe(III), and orange is Fe(II).

Among fungi, iron acquisition mechanisms are most explored in *Saccharomyces cerevisiae* where, in addition to the siderophore transporters, several other transporters are involved in iron uptake (**Figure 1**) 26. One of the mechanisms present in yeast is the reduction of Fe^3+^, which is performed by membrane-bound metalloreductases 27 followed by the transport of Fe^2+^ across the cell membrane by high-affinity or low-affinity iron transport systems. At low iron levels, the high-affinity iron transport system and the siderophore transport system are working. Two components of the high-affinity iron transport system are FTR1 permease and FET3 copper-dependent Fe^2+^ oxidase 24. In low iron environment, many bacteria and fungi secrete low-molecular weight ferric iron chelators called siderophores 23,25. While many ascomycetous fungi produce hydroxamate siderophores, this ability is not known for Mucorales fungi. Instead, they secrete a polycarboxylate siderophore, rhizoferrin 25. Although Mucorales fungi secrete only rhizoferrin, they can utilize hydroxamate siderophores as xenosiderophores. For example, deferoxamine is a bacterial siderophore, which was used in clinical practice as an iron-chelating agent to treat iron overload. This treatment is one of the most significant risk factors for mucormycosis 28. Other iron-chelating agents, such as deferiprone or deferasirox, which lack the xenosiderophore activity for *Rhizopus*, can cause an iron-starvation for the fungus 29,30. Sit1 (also called ARN3) encodes a ferrichrome and ferrioxamine transporter, which belongs to the SIT subfamily of the major facilitator superfamily (MFS). In many fungi, such as *S. cerevisiae*, *Candida albicans*, *Candida glabrata*, *Cryptococcus neoformans* and *Aspergillus fumigatus*, hydroxamate type siderophore-iron chelates are taken up through Sit1/ARN3 transporters 31.

Little is known about the siderophore transporters in Mucorales fungi. Usage of hydroxamate siderophores (e.g., deferoxamine) as xenosiderophores can play a role in the development of mucormycosis 28,32. *Mucor lusitanicus* is a widely used model organism for studying various molecular microbiological issues in Mucorales 33,34,35,36,37,38,39,40,41,42,43,44, including the pathomechanisms of mucormycosis-causing fungi 42,44. The genome of *M. lusitanicus* contains one siderophore-iron (ferrioxamine):H^+^ symporter gene (*sit1*) belonging to the SIT subfamily of MFS transporters. In this study, transcription of this gene was examined, and its function was analyzed using knockout mutants.

## RESULTS 

### *In silico* analysis and characterization of the Sit1 protein and relative transcript level of the *sit1 *gene 

We evaluated the specificity of the siderophore transporter HMM profile we constructed using annotated genomes from nine fungal species, which were downloaded from the NCBI Datasets database. The species included in the analysis were *Aspergillus nidulans* FGSC A4 (Acc. no. GCF_000011425.1), *A. fumigatus* Af293 (Acc. no. GCF_000002655.1), *Fusarium oxysporum* Fo47 (Acc. no. GCF_013085055.1), *Fusarium graminearum* PH-1 (Acc. no. GCF_000240135.3), *Alternaria alternata* SRC1lrK2f (Acc. no. GCF_001642055.1), *Exserohilum turcica* Et28A (Acc. no. GCF_000359705.1), *Schizosaccharomyces pombe* I-540 (Acc. no. GCA_043157545.1), *S. cerevisiae* S288C (Acc. no. GCF_000146045.2), and *M. lusitanicus* CBS 277.49 (Acc. no. GCA_040256725.1). The annotation of *M. lusitanicus* used in this study corresponds to version 3 available on the MycoCosm web portal (https://mycocosm.jgi.doe.gov/mycocosm/home) 45. During the evaluation of the hmmsearch results, we considered only those hits with a bit score higher than 400. All hits meeting this criterion were further verified using the TCDB database, which confirmed without exception that the identified proteins were siderophore transporters. The sequences and the bit score values of the identified siderophore transporters are provided in Supplementary Table S1. In the *M. lusitanicus* CBS 277.49 genome, we found a total of eight siderophore transporters, which corresponded to the siderophore transporters annotated in the MycoCosm portal. Subsequently, we conducted two PSI-BLAST searches using the protein sequence of interest (Acc. no. KAL0139362.1), one with default parameters and another restricted to Ascomycota species. The results of both searches were combined, and after removing duplicates and reducing the dataset with the Treemmer script, we performed phylogenetic reconstruction. Since the majority of hits were annotated as hypothetical proteins or proteins containing MFS domains, we validated these sequences using our HMM profile, confirming that all were indeed siderophore transporters. The dataset was further supplemented with sequences published by Aguiar *et al*. (2021) 46. The results of the maximum likelihood analysis also confirmed that the protein under investigation is a siderophore

transporter, as it clustered with previously annotated siderophore transporters, and its sister clade also contained several annotated siderophore transporters (Supplementary Figure S1). Among these transporters in the *M. lusitanicus* genome database (https://mycocosm.jgi.doe.gov/Mucci3/Mucci3.home.html), one

potential Sit1 protein coding gene (protein ID: 1388546) was found. The putative Sit1 transporter protein consists of 606 amino acids and contains the characteristic motif of the MFS transporters between the 93-512 amino acids. It also contains 14 transmembrane helices (Supplementary Figure S2A). The predicted tertial structure also supports the existence of transmembrane helices and the channel they form (Supplementary Figure S2B). Ramachandran plot analysis indicated that 93.66% and 2.34% of the residues are located in the favored and allowed regions.

**Figure 2:**
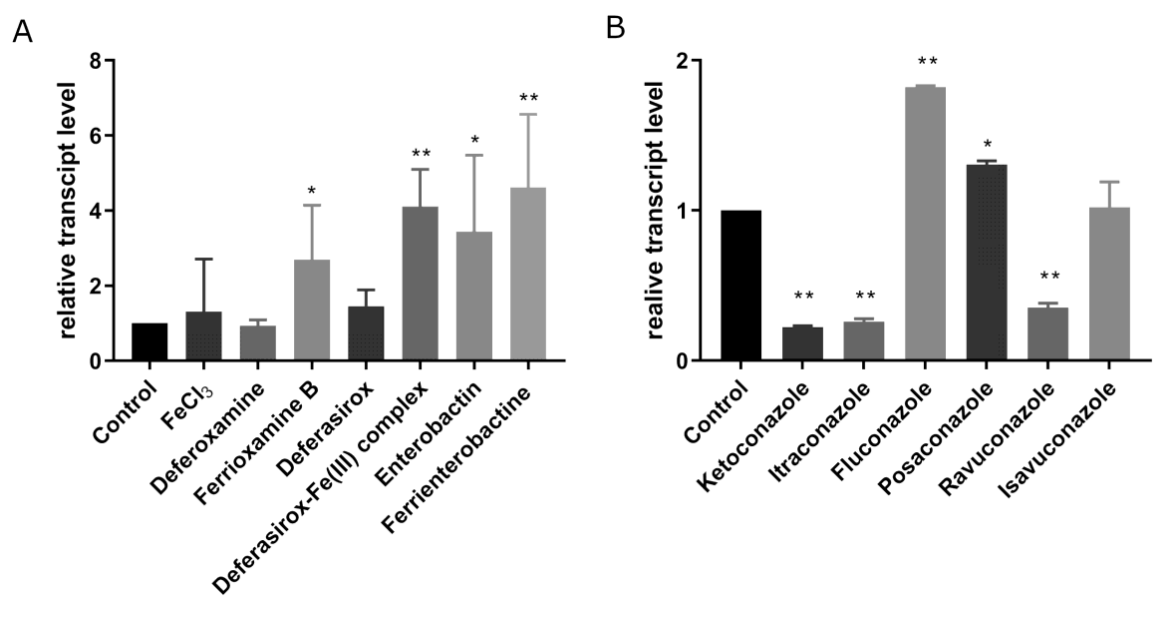
FIGURE 2: Relative transcript levels of the *sit1* gene of the *M. lusitanicus* MS12 strain after treatment of iron and iron chelators (A) and azoles (B). Relative transcript level of *sit1* gene was analyzed via quantitative reverse transcription PCR. MS12 was grown in liquid RPMI-1640 medium for two days at 25°C; transcript level of each gene measured in the untreated control was taken as 1. The relative quantification of the gene expression was achieved with the 2^-ΔΔCt^ method using the actin gene of *M. lusitanicus* as a reference. The presented values are averages of three independent experiments of two independent isolates; error bars indicate standard deviation. Relative transcript values followed by * and ** significantly differed from the untreated control according to the paired t-test (*p < 0.05 and **p < 0.01).

Relative transcript level of the *sit1* gene significantly increased after the treatment of the fungus with ferrioxamine B, deferasirox-Fe(III) complex, enterobactin and ferrienterobactine (**Figure 2A**). In azole resistant clinical strains of *A. fumigatus *and *S. cerevisiae* siderophore transporter genes are often downregulated 47,48,49, therefore relative transcription of *sit1* was also examined after azole treatment. Transcript level of *sit1* significantly increased after fluconazole (p<0.01) and posaconazole (p<0.05) treatment, while ketoconazole and itraconazole resulted in decreased transcript levels (**Figure 2B**).

### Relative transcript level of other iron acquisition genes in the *sit1*Δ strain

Two *sit1* mutants (*sit1*Δ/1 and *sit1*Δ/2) were obtained by transformation and homologous recombination with the *pyrG* cassette flanked with sequences 5′ and 3′ end of the *sit1* gene ORF (Supplementary Figure S3). A total of eleven *pyrG*^+^ transformants were obtained, which were grown on selective medium for several vegetative cycles to obtain homokaryotic transformants (Supplementary Table S3).

**Figure 3:**
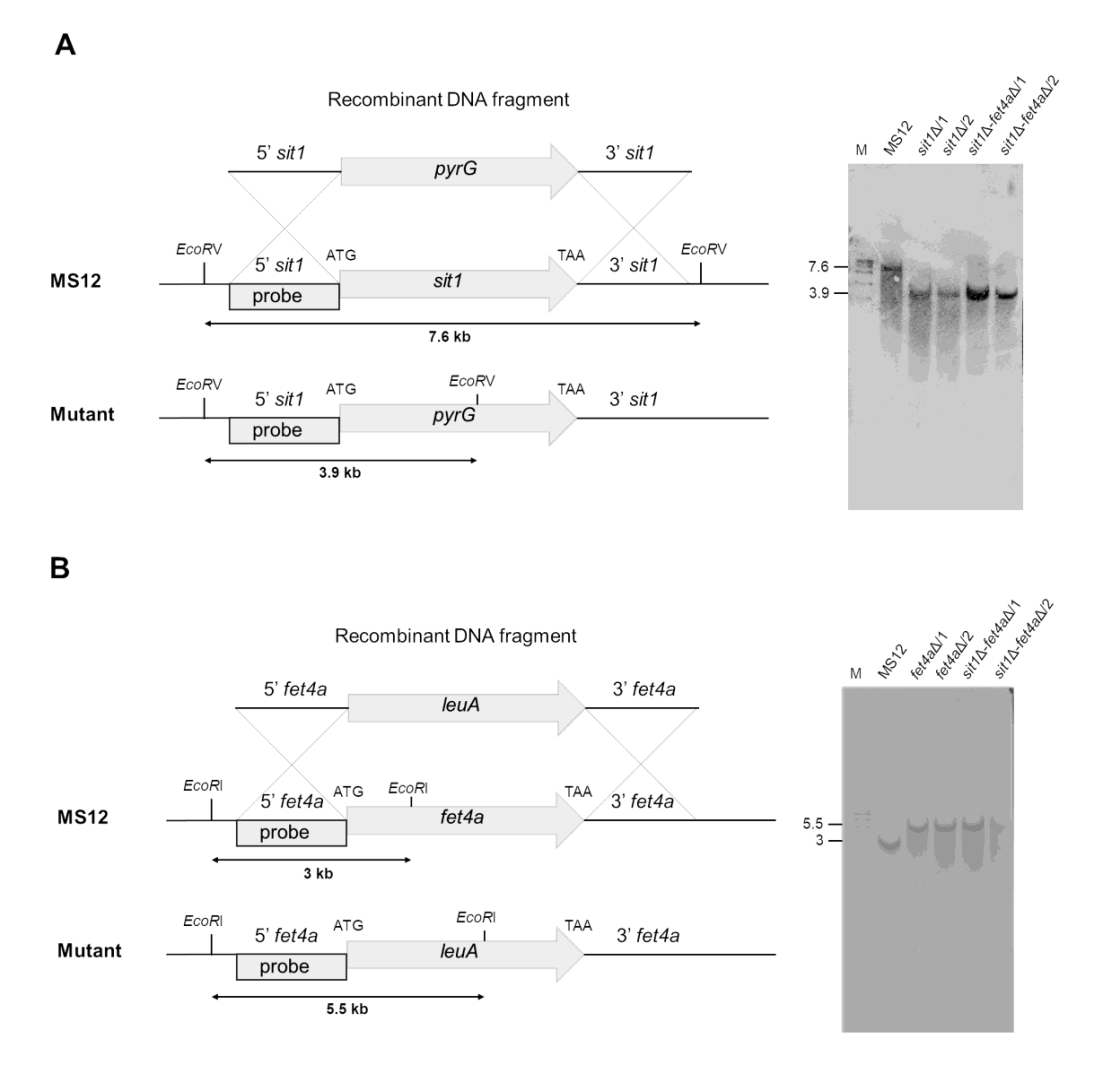
FIGURE 3: Deletion of *sit1* and *fet4a* in *M. lusitanicus* confirmed by Southern Blot (A) *sit1* (B) *fet4a*. The 5′ and 3′ regions upstream and downstream from the start and stop codons, respectively, of *sit1* or *fet4a* were designed to flank the *pyrG* or *leuA* selection marker for gene deletion (A and B). For each diagram, the recombinant fragments that were used to transform protoplasts of the strain MS12 (*pyrG*^⁻^, *leuA*^⁻^) are shown. Molecular confirmation by Southern blotting was performed using specific probes for each gene. DNA samples from transformed and parental (MS12) strains were digested with the indicated restriction enzymes.

The homologous replacement was confirmed through PCR (Supplementary Figure S4) and Southern blot hybridization (**Figure 3**) with a probe homologous to the 5’ of *sit1* gene that can discriminate between wild-type and mutant alleles (**Figure 3**).

**Figure 4:**
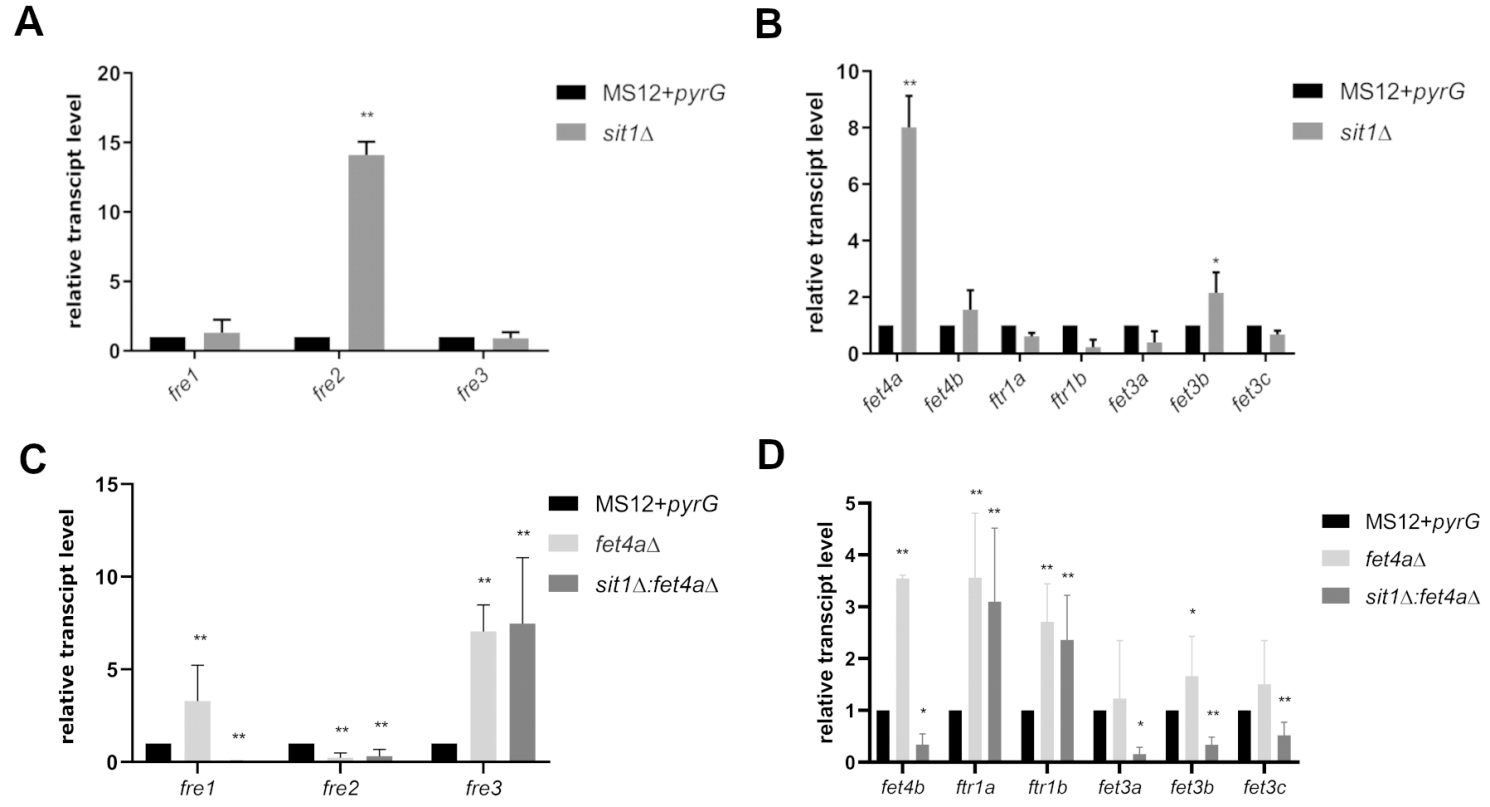
FIGURE 4: Relative transcript level of the iron acquisition related genes in the *sit1*Δ (A and B), *fet4a*Δ (C and D), and *sit1*Δ:*fet4a*Δ (C and D) strains compared to the MS12+*pyrG* strain. Relative transcript level of iron acquisition related genes was analyzed via quantitative reverse transcription PCR. The relative quantification of the gene expression was achieved with the 2^-ΔΔCt^ method using the actin gene of *M. lusitanicus *as a reference. The transcript level of each gene measured in MS12+*pyrG* strain was taken as 1. The presented values are averages of three independent experiments of two independent isolates; error bars indicate standard deviation. Relative transcript values followed by * and ** significantly differed from the untreated control according to the unpaired t-test (*p<0.05, **p < 0.01).

Expression of the genes of the low- and the high-affinity iron transport systems were examined in the *sit1* knockout mutant. Ferric reductases exhibit some siderophore reductase activity and they can reduce and release the siderophore-bound iron 50. Blast search using the *S. cerevisiae* *FRE1* sequence (P32791) found three putative ferric reductase coding genes, Fre1, Fre2, and Fre3, (protein IDs: 1474878; 1527270; 1557900) with in the *M. lusitanicus* genome. Among them, *fre2* was found to be significantly upregulated in the *sit1*Δ strain (**Figure 4A**).

The two components of the *S. cerevisiae* high-affinity iron transport system are FTR1 permease and FET3 copper-dependent Fe^2+^ oxidase 24. Homologous sequences of the high and low affinity iron uptake system genes were searched on the *M. lusitanicus* genome using the *S. cerevisiae* *FET4* (YMR319C), *FTR1* (P40088) and *FET3* (YMR058W) sequences. Genes of two putative low affinity iron permeases, Fet4a (protein ID: 1364625) and Fet4b (protein ID: 1546754), two Ftr1 family iron permeases, Ftr1a (protein ID:1533506) and Ftr1b (protein ID:1461375) and three copper dependent iron oxidases Fet3a (protein ID:1559204), Fet3b (protein ID: 1533515), and Fet3c (protein ID:1516145) were found, respectively. In the *sit1*Δ strain, two genes, a low affinity iron permease (*fet4a*, 1364625) and a copper dependent iron oxidase (*fet3b*, 1533515), were significantly upregulated (**Figure 4B**).

### Relative transcript levels of other iron acquisition genes in the *fet4a*Δ and *sit1*Δ:*fet4a*Δ strains

Two *fet4a* mutants (*fet4a*Δ/1 and *fet4a*Δ/2), and two *sit1*:*fet4a* (*sit1*Δ:*fet4a*Δ/1, *sit1*Δ:*fet4a*Δ/2) double knockout mutants were obtained by transformation and homologous recombination with the *leuA* cassette flanked with sequences 5′ and 3′ end of the *fet4a* gene ORF (Supplementary Figure S3B). To create a double knockout mutant, the previously created *sit1*Δ was used in transformation experiment. A total of nine *leuA*^+^ and 14 *pyrG*^+^:*leuA*^+^ transformants were obtained, which were grown on selective medium for several vegetative cycles to obtain homokaryotic transformants (Supplementary Table S3).

The homologous replacement was confirmed through PCR (Supplementary Figure S5) and Southern blot hybridization (**Figure 3**) with a probe homologous to the 5’ of *fet4a* gene that can discriminate between wild-type and mutant alleles (**Figure 3**).

Among the ferric reductase coding genes, the lack of the *fet4a* in *fet4a*Δ, resulted in increased transcript levels for *fre1 *and* fre3* (**Figure 4C**), while the simultaneous deletion of *fet4a* and *sit1* in *sit1*Δ:*fet4a*Δ increased the transcript level of the *fre3* gene only (**Figure 4C **and** D**). Relative transcript levels of *fet4b*, *ftr1a*, *ftr1b*, and *fet3b* significantly increased in the *fet4a*Δ mutant. At the same time, *ftr1a* and *ftr1b* showed significantly increased transcript levels in *sit1*Δ:*fet4a*Δ (**Figure 4D**).

### Ferric reducing capacity and quantification of deferoxamine in liquid media of the *sit1*Δ strain

**Figure 5:**
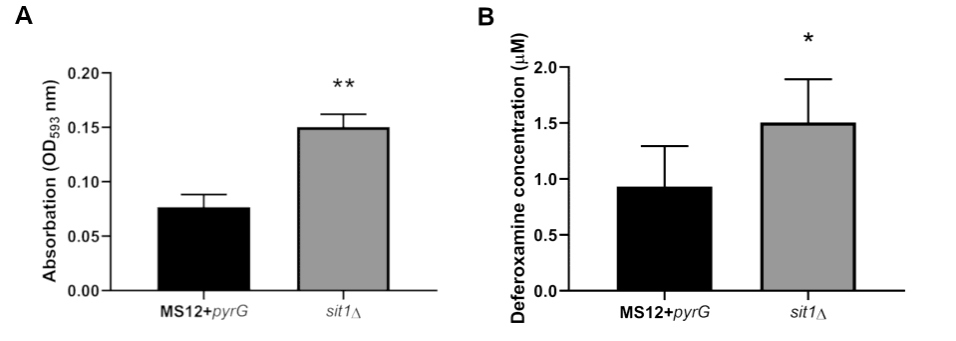
FIGURE 5: Determination of ferric reducing capacity (A) and quantification of deferoxamine in liquid media (B) of the *sit1*Δ strain compared to the MS12+*pyrG* strain. To assess ferric reducing capacity, a FRAP assay was performed using fungal strains pre-cultivated for one day in iron-free Czapek-Dox medium (10^⁶^ spores/mL). After adding FRAP reagent, absorbance was measured at 593 nm. For deferoxamine quantification, the fungus was pre-cultivated, then treated with 10 µM ferrioxamine B for 24 hours. Fungal biomass was removed via centrifugation and filtration. Deferoxamine levels in the medium were measured using LC-HRMS system using PRM method targeting the specific m/z of deferoxamine. The presented values are averages of three independent experiments of two independent isolates; error bars indicate standard deviation. Relative transcript values followed by * and ** significantly differed from the untreated control according to the unpaired t-test (*p < 0.05, **p < 0.01).

We performed a ferric reducing assay (FRAP) using the *sit1*Δ mutant strain, which showed significantly higher iron-reducing activity compared to the control strain (**Figure 5A**). This is likely due to the increased expression of iron reductase observed in the RT-qPCR analysis. Furthermore, in liquid Czapek-Dox medium, we performed LC-HRMS analysis to measure the amount of deferoxamine following the addition of ferrioxamine B after one day of iron starvation. The mutant strain liberated significantly more deferoxamine than the control, suggesting enhanced iron reduction (**Figure 5B**). These results support the hypothesis that, in the absence of the Sit1 transporter, the fungus utilizes an alternative iron acquisition pathway involving a reductase enzyme. Siderophore secretion was also examined via CAS agar. Around the colony of MS12+*pyrG* strain there is a strong, orange band which is absent in the *sit1*Δ mutant (Figure S6).

### Growth ability of the mutant strains 

**Figure 6:**
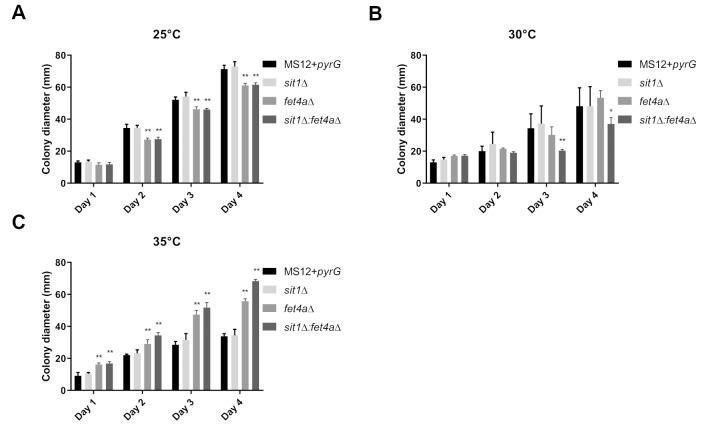
FIGURE 6: Colony diameters of the *sit1*Δ, *fet4a*Δ single- and *sit1*Δ:*fet4a*Δ double knockout mutants and the control MS12-*pyrG* strain on YNB medium at 25°C (A); 30°C (B); 35°C (C). The presented values are averages; colony diameters were measured during three independent cultivations of two independent isolates (error bars indicate standard deviation). Values followed by ** significantly differed from the corresponding value of the MS12-*pyrG* strain according to the two-way Anova (p < 0.01).

Growth ability of the *sit1*Δ, *fet4a*Δ and *sit1*Δ:*fet4a*Δ strains were analyzed at different temperatures (i.e., 25, 30 and 35°C) on YNB medium. Colony size of *sit1*Δ did not differ significantly from that of the control MS12+*pyrG* strain. Pathogenic fungi grow at higher temperatures (up to 37°C) compared to laboratory organisms which grow at room temperature; therefore, we examined the growth at different temperatures to investigate the effects of heat stress. *fet4a*Δ and* sit1*Δ:*fet4a*Δ showed significantly decreased growth on the second, third and fourth day at 25°C. At 30°C, the colony diameter of *sit1*Δ:*fet4a*Δ was significantly decreased on the third and fourth day. Interestingly, *fet4a*Δ and showed significantly increased growth ability at 35°C compared to the control (**Figure 6**).

**Figure 7:**
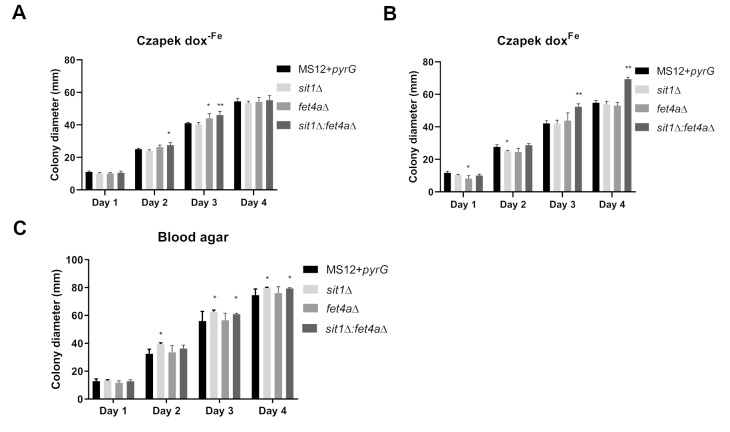
FIGURE 7: Colony diameters of the *sit1*Δ, *fet4a*Δ and *sit1*Δ:*fet4a*Δ mutants and the control MS12-*pyrG* strain at 25 °C on Blood agar (A) Czapek-Dox agar with (B) and without (C) FeCl_3_. The presented values are averages; colony diameters were measured during three independent cultivations of two independent isolates (error bars indicate standard deviation). Values followed by ** significantly differed from the corresponding value of the MS12-*pyrG* strain according to the two-way Anova (p < 0.01).

Colony diameter was also measured on blood and Czapek-Dox agar with (Czapek-Dox^Fe^) or without iron (Czapek-Dox^-Fe^) to observe the growth in the absence of free iron and under iron limited conditions (**Figure 7**). The use of different culture media contributes to a better understanding of the functional role of the examined genes. On iron-free Czapek-Dox medium, siderophore production is essential for growth, as it facilitates iron acquisition under iron-limited conditions. In contrast, iron-supplemented Czapek-Dox medium allows assessment of alternative iron uptake mechanisms that may compensate for the absence of the *sit1* gene. Blood-supplemented medium contains complex iron sources, which mimic host-like environments and can reveal whether Sit1 contributes to the utilization of biologically relevant iron forms.

The *sit1*Δ did not differ significantly from the control on Czapek-Dox^-Fe^, while *fet4a*Δ exhibited increased growth on the third day and the *sit1*Δ:*fet4a*Δ strain showed significantly increased growth on the second and third day (**Figure 7A**). On Czapek-Dox^Fe^ agar the *sit1*Δ:*fet4a*Δ showed significantly increased growth on the third and fourth day (**Figure 7B**). On blood agar the *sit1*Δ and* sit1*Δ:*fet4a*Δ strains showed significantly increased growth, while the *fet4a*Δ did not differ significantly from the control (**Figure 7C**).

Sensitivities of the *sit1*Δ mutants to different azoles and AmB were determined by using a broth microdilution assay, and the MICs of the antifungal agents were determined (Supplementary Table S4). Lack of the *sit1* gene slightly affected the sensitivity of the fungus to posaconazole, ravuconazole, izavuconazole and amphotericin B as the mutant isolates showed weaker growth after treatments with these drugs (Supplementary Table S4).

### Knockout of the *sit1* and *fet4a* genes affects the germination ability of the sporangiospores

**Figure 8:**
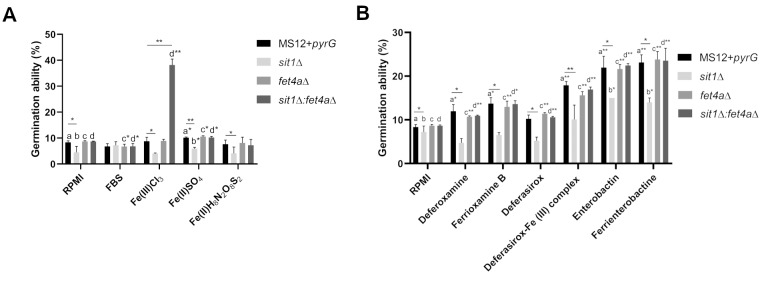
FIGURE 8: Percentage of the germinating sporangiospores of the created mutant isolates using different iron compounds (A) and siderophores and siderophore-iron complexes (B). Germ tube development was counted after cultivations for 3.5 h at 25°C. The presented values are averages of three independent experiments of two independent isolates (error bars indicate standard deviation). Values followed by * and ** significantly differed from the corresponding value of the MS12 strain according to the paired t-test and unpaired t-test (*p < 0.05, **p < 0.01).

Sporangiospores were inoculated in liquid RPMI-1640 medium supplemented with FBS, FeCl_3_, FeSO_4_, and FeH_8_N_2_O_8_S_2 _(**Figure 8A**). The *sit1*Δ mutant showed decreased germination in RPMI-1640 compared to the control MS12+*pyrG *strain. In FBS-supplemented RPMI medium, both *fet4a* Δ and *sit1*Δ:*fet4a*Δ showed decreased germination compared to the control culture in RPMI-1640 (**Figure 8A**). In the presence of FeCl_3_, *sit1*Δ showed decreased germination compared to the MS12+*pyrG* control, while *sit1*Δ:*fet4a*Δ showed significantly increased germination (**Figure 8A**). FeSO_4 _increased the germination of all strains, except of *sit1*Δ, which showed decreased germination compared to the control MS12+*pyrG *(**Figure 8A**). In liquid RPMI-1640 supplemented with FeH_8_N_2_O_8_S_2_, the *sit1*Δ mutant showed decreased germination compared to the MS12+*pyrG *control strain (**Figure 8**).

Deferoxamine and ferrioxamine B increased the germination in all strains compared to the cultivation in RPMI-1640, except in the case of *sit1*Δ, which showed decreased germination compared to the MS12+*pyrG* control (**Figure 8B**). Deferasirox increased the germination of *fet4a*Δ and *sit1*Δ:*fet4a*Δ strains compared to the control cultivation in RPMI-1640, while *sit1*Δ had significantly decreased germination compared to the MS12+*pyrG* control (**Figure 8B**). Deferasirox-Fe (III) complex increased the germination of MS12+*pyrG*, *fet4a*Δ and *sit1*Δ:*fet4a*Δ strains compared to their germination in RPMI-1640, while *sit1*Δ and *fet4a*Δ showed decreased germination compared to the MS12+*pyrG* control (**Figure 8B**). Enterobactin and ferrienterobactine increased germination capacity of all strains (**Figure 8B**). Interestingly *sit1*Δ had decreased germination compared to the MS12+*pyrG* control in both media (**Figure 8B**). These results suggest that the germination ability of *sit1*Δ is significantly reduced due to the lack of the *sit1 *gene.

### *In vivo* virulence of the *sit1*Δ, *fet4a*Δ and *sit1*Δ:*fet4a*Δ mutants

**Figure 9:**
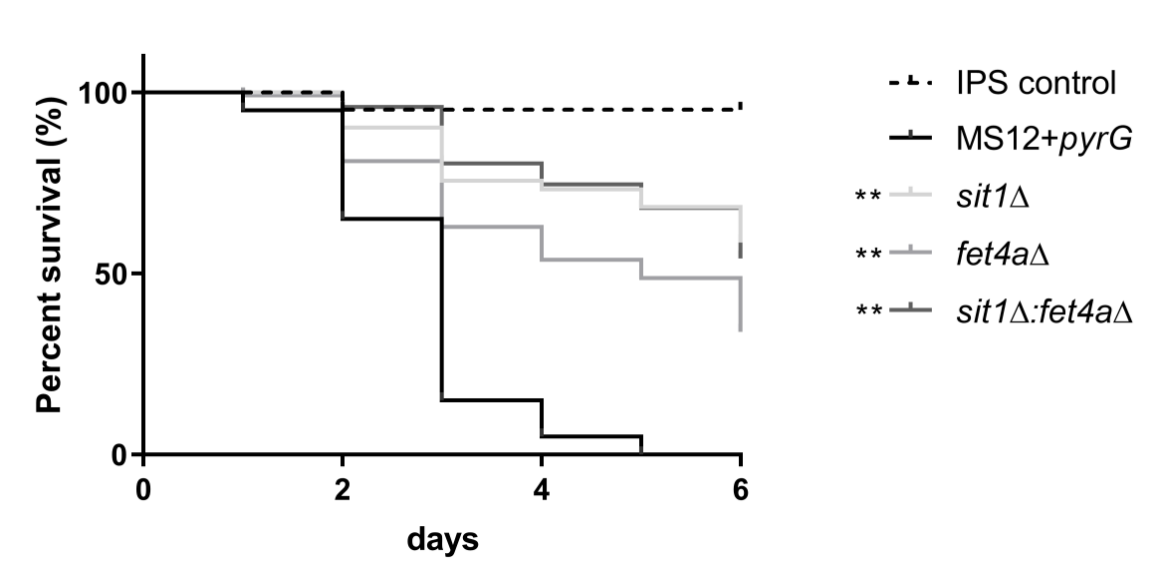
FIGURE 9: Survival of *G. mellonella* (n=20) infected with the MS12+*pyrG*, *sit1*Δ, *fet4a*Δ and *sit1*Δ:*fet4a*Δ strains. The presented values are averages; survival curves were determined from three independent cultivations of two independent isolates. Survival curve followed by asterisks were significantly differed from the control strain according to the Log-rank (Mantel-Cox) test (**p <=0.01).

To investigate the role of Sit1 and the Fet4a proteins in the pathogenicity of *M. lusitanicus*, wax moth larvae (*Galleria mellonella*) were used as a non-vertebrate animal model for *in vivo* virulence studies. Twenty larvae were infected with each *M. lusitanicus* isolate. The results shown are representatives of at least three independent experiments. In the *Galleria *model (**Figure 9**), knockout of *sit1* and *fet4a* resulted in significantly (**p<0.01) decreased virulence compared to the control strain.

## DISCUSSION

Microbes utilize various types of siderophores using different transport systems and several microorganisms can utilize and transport xenosiderophores as well, that are produced by other microorganisms. Although *S. cerevisiae* cannot synthesize siderophores, it has four siderophore transport systems with different substrate specificities, such as Arn1 (ferrichrome transporter), Arn2/Taf1 (TAFC transporter), Arn3/Sit1 (hydroxamate siderophore transporter) and Arn4/Ernb (enterobactin transporter) 31,51. In the genome of *M. lusitanicus*, one gene (*sit1*) for a putative Arn3/Sit1 transporter was found.

Siderophore transporters belong to the MFS. The catalyzed transport is an energy-dependent and highly specific process 52. Arn3/Sit1 plays a role in ferrichrome and ferrioxamine transport in *S. cerevisiae, C. albicans*, *C. glabrata*, *C. neoformans*, and *A. fumigatus* 31. Siderophore transporters from* S. cerevisiae* have 15 transmembrane domains (TMs), while the number of predicted TMs in *A. fumigatus* and *C. glabrata* Sit1 are 14 with cytosolic N- and C-termini 46,52,53. The putative Sit1 protein of *M. lusitanicus* has 14 predicted TMs. N- and C- termini of the putative protein seem to be cytosolic, which agrees with the predicted lack of a signal sequence.

Expression of this gene significantly increased after fluconazole and posaconazole treatment and significantly decreased after ketoconazole, itraconazole, and ravuconazole treatment. In azole resistant organisms, expression of siderophore transporter genes is reduced in some cases but the absence of the siderophore transporter gene does not affect sensitivity to azoles 46,47. In a pan-azole-resistant clinical *A. fumigatus*, the expression of siderophore transporter *mirB* is significantly decreased 47. In fluconazole and itraconazole resistant *S. cerevisiae*, *sit1* was down-regulated 49, but the absence of this gene did not affect sensitivity to azoles 48. Furthermore, iron transporter genes were down-regulated in fluconazole-resistant clinical *C. albicans* isolates as well 49. The lack of *sit1* gene had no effect on susceptibility of mutant isolates to the tested antifungal agents in *M. lusitanicus*.

In the past, deferoxamine therapy proved to be a major risk factor for mucormycosis 54 showing the importance of iron acquisition in the virulence of Mucorales. Hemodialysis patients receiving deferoxamine to treat iron overload were uniquely predisposed to disseminated mucormycosis, but the modern iron-chelating agents, such as deferiprone or deferasirox, lack a xenosiderophore activity for *Rhizopus* and can cause an iron-starvation effect for the fungus 29. In our study, ferrioxamine B, deferasirox-Fe(III) complex, enterobactin, ferrienterobactine increased the relative transcript level of *sit1* in *M. lusitanicus.*

Iron release from xenosiderophores can occur via two main mechanisms: either through extracellular reduction by iron reductases or via intracellular hydrolytic degradation following uptake. 55 Our results support the role of Sit1 as a siderophore transporter. Upon addition of ferrioxamine B, the *sit1*Δ strain showed significantly elevated iron-reducing activity in the FRAP assay and a significantly higher concentration of deferoxamine could be detected in the liquid medium compared to the control strain. These findings suggest that the absence of Sit1-mediated uptake leads to the fungus relying more on extracellular reduction to access iron from ferrioxamine B.

Deferoxamine, deferasirox and deferasirox-Fe(III) complexes increased the germination ability of the control MS12+*pyrG* strain, however the lack of *sit1* did not influence the germination of the *sit1*Δ strain in the presence of siderophore. Interestingly the lack of *fet4a *and *sit1* increased the germination capacity of mutant strains in deferoxamine and ferrioxamine B complex supplemented media compared to RPMI medium without siderophore. In *S. cerevisiae, *the lack of Sit1 had no effect on the uptake of ferrioxamine as an iron source, however the simultaneous deletion of *Sit1* and *FET3* resulted in no growth on ferrioxamine 56. In *A. fumigatus*, Sit1 is essential for utilization of ferrioxamine-type xenosiderophores ferrioxamine B, G, and E 46.

Enterobactin is a catecholate-type siderophore which is produced primarily from Gram-negative bacteria such as *Escherichia coli*, *Salmonella typhimurium*, *Klebsiella pneumoniae* etc. Previously, it was revealed that *A. fumigatus*,* S. cerevisiae*, *C. albicans*, and *C. glabrata* cannot utilize it 46. In our study, enterobactin increased the germination ability of *M. lusitanicus* regardless of the absence of the *sit1 *and/or *fet4a* genes. However, as we mentioned above, the relative transcript level of *sit1* significantly increased after enterobactin or ferrienterobactine treatment. This finding, i.e., the presence of siderophores enhances the transcription regardless of their chemical structures, suggests that the regulation of genes involved in iron uptake, or at least siderophore uptake, is coordinated at the level of transcription in *Mucor*. Co-infection of mucormycosis with bacteria is a very rare occurrence, however some cases have already been described 57,58. Choi *et al*. 57 followed up 61 patients with mucormycosis between 2009 and 2019. A total of 24 patients were found to have bacterial co-infection with mucormycosis, in five of them *K. pneumoniae* were isolated. Furthermore, some *Rhizopus* species have been found to harbour *K. pneumoniae* as endosymbiotic bacteria 59, but the effect of the entrobactin on the growth of the Mucorales has not yet been studied.

In *S. cerevisiae*, iron homeostasis is highly regulated at the transcriptional level by the iron responsive transcriptional activators Aft1 and Aft2, which control the iron regulatory genes, including *FET3*, *FTR1*, *FRE1*, *FRE2*, *ARN1-4*, *FIT1-3*, *FET5*, and *FTH1*, involved in iron acquisition and intracellular iron distribution 60. Our results suggest that this may work similarly in *M. lusitanicus*, as knocking out the *sit1* gene increased the transcriptional activity of the genes encoding an iron reductase (Fre), a low-affinity iron permease (Fet4) and a copper-dependent Fe^2+^ oxidase (Fet3) also indicating the co-regulation of these genes. The genome of *M. lusitanicus* encodes two Fet4 proteins (Fet4a and Fet4b). Knockout of *fet4a*, which is upregulated after *sit1* knockout, resulted in increased transcript level of the *fet4b* gene. Apparently, *fet4b* can compensate to some extent for the lack of *fet4a*. This compensatory effect was also observed in cases of other paralogous genes of *Mucor*, such as *hmgR*, *pdr*, or *egr6* 44,61,62.

Our results suggested that the lack of *sit1* resulted in an increased transcript level of ferric reductase gene (*fre3*), and genes of low-(*fet4b*), and high-affinity iron (*fet3b*) transporter system. The lack of *fet4a* increased the transcript level of the ferric reductase genes (*fre1* and *fre3*) and high-affinity (*fet4b*, *ftr1a,* *ftr1b*, and *fet3b*) iron transporter gene *ftr1*. Our result suggest that Fet4a is the dominant member of low affinity iron transporter system because its deletion results in significantly increased transcript level of *fet4b. *Furthermore, simultaneous deletion of *sit1* (siderophore transporter) and *fet4a* (high-affinity iron transporter) genes resulted in an increased transcript of *ftr1* genes as high-affinity iron transport genes. The results indicate that the siderophore transporter system, low-affinity iron transporter system and high-affinity transporter system are working together and in the absence of one the fungus tries to take up iron by other pathways. However, homology search did not produce any hits with either Aft1 or Aft2 in the *M. lusitanicus* genome, we must assume that these genes are regulated by other factors in* Mucor*.

Our results suggest that the lack of *sit1* may influence the germination of *Mucor* in the presence of siderophores or siderophore-iron complexes. The germination ability of MS12-*pyrG*,* fet4a*Δ and *sit1*Δ:* fet4a*Δ strains significantly increased in the presence of siderophores and siderophore-iron complexes. Increased germination ability due to the siderophores or siderophore-iron complexes may play a role in the pathogenicity of Mucorales. We examined the function of *sit1* in the pathogenesis of *M. lusitanicus *using the non-vertebrate *G. mellonella* model. The *sit1*Δ mutant showed altered germination and growth ability under the tested conditions and virulence of this mutant significantly decreased, however the* fet4a*Δ and *sit1*Δ*:fet4a*Δ did not show altered germination, only decreased growth ability, yet these mutants still had decreased virulence. In the case of *C. glabrata*, the lack of *sit1* resulted in reduced survival of the fungus within the macrophages 53,63. In *C. albicans*, Sit1 was required for the invasion of human epithelial cells *in vitro*, while it was not essential for systemic infection 64. In case of *A. fumigatus*, Sit1 and Sit2 were not required for virulence, but the lack of both encoding genes resulted in higher conidial killing activity of neutrophil- and macrophage-like cells 65. Understanding the siderophore uptake system of pathogenic fungi is important, due to several biotechnological applications, potential in treatment and diagnostics. Siderophores can be combined with antibiotic and used as a ‘Trojan horse’ for targeted drug delivery. Furthermore, recent discoveries have identified iron acquisition as a key virulence factor in mucormycosis, and novel therapeutic approaches such as antibodies targeting fungal iron transporters offer promising potential to improve treatment outcomes 16,66.

The *M. lusitanicus* genome contains one Sit1, siderophore transporter, encoding gene. Characterization of the *sit1* knockout mutant showed Sit1 is important for normal spore germination and virulence of *M. lusitanicus*, furthermore it plays an important role in the ferrioxiamine B uptake. Our results further show that enterobactin can increase the germination ability of *Mucor* and its uptake is independent from Sit1. It is also clear that the iron uptake systems are interconnected and presumably under common control and the main low-affinity iron transporter is Fet4a.

## MATERIAL AND METHODS

### Strains, media and growth conditions 

*Mucor lusitanicus* strain MS12, a double auxotrophic (*leuA*- and *pyrG*-) derivative of the wild-type strain CBS 277.49 67 and MS12+*pyrG *68, in which the uracil auxotrophy had been complemented, were used in the study. For nucleic acid extraction from *Mucor*, 10^6^ sporangiospores were plated onto a solid minimal medium (YNB; 10 g glucose, 0.5 g yeast nitrogen base without amino acids [Sigma Aldrich, St. Louis, MO, USA], 1.5 g (NH_4_)_2_SO_4_, 1.5 g sodium glutamate and 20 g agar per liter) supplemented with leucine and/or uracil (0.5 mg/ml), if required. In some cases, RNA extraction was performed after cultivation in 30 mL RPMI-1640 without agar (Biosera, Kansas City, MO, USA); in this case, the inoculum size was 10^4^ sporangiospores/mL. Fungal cultures were grown for four days under continuous light at 25°C. To test the effect of azoles on the gene expression, strains were grown in 10 mL liquid RPMI-1640 containing ketoconazole (Alfa Aesar, Haverhill, MA, USA), itraconazole (Across Organics, Geel, Belgium) and fluconazole (Alfa Aesar, Haverhill, MA, USA) in final concentrations of 8 μg/mL, while the final concentration of ravuconazole (Sigma Aldrich, St. Louis, MO, USA), posaconazole (Sigma Aldrich, St. Louis, MO, USA) and isavuconazole (Sigma Aldrich, St. Louis, MO, USA) was 2 μg/mL. Anaerobic growth was performed in a BBL GasPak Anaerobic System (Becton Dickinson, Franklin Lakes, NJ, USA) at 25°C. For the antifungal susceptibility test, *Candida krusei* ATCC 6258 was used as the reference strain. To test the effect of FeCl_3_, deferoxamine, ferrioxamine B, deferasirox, deferasirox-Fe(III) complex, enterobactin and ferrienterobactine on gene expression, 10^5^ spores/mL were inoculated in RPMI-1640 containing 10 µM of chemical.

To measure the colony diameters of the strains, YNB, Czapek-Dox agar (2 g NaNO_3_, 0,5 g MgSO_4_ x 7H_2_O, 0,5 g KCl, 0,5 g K_2_HPO_4_ 30 g saccharose, 10 mg ZnSO_4_ x 7H_2_O, 10 mg FeSO_4_ x 7H_2_O, 3 mg CuSO_4 _, 30 g agar per liter) and Blood agar (4 g peptone, 10 g glucose, 9 g NaCl, 20g agar and 50 mL defibrinated sheep blood [Hemoking Ltd., Mezőhegyes, Hungary] per liter) were used. Onto the centre of the solid medium, 10^4 ^sporangiospores were inoculated. The diameter of the colonies was measured daily after incubating the plates at 25, 30, and 35°C using the MS12+*pyrG* strain as the growth control. In each case, colony diameters of two colonies were measured using three biological and two technical replicates.

One liter of CAS blue agar was made using 60,5 mg CAS dissolved in 50 mL distilled water and mixed with 10 mL of iron solution (1 mM Fe(III)Cl .6 H_2_O , 10 mM HCI). Under stirring, this solution was slowly added to 72.9 mg HDTMA (Hexa decyl tri methyl-ammonium bromide) dissolved in 40 mL water then autoclaved. A mixture of 750 mL water, 15 g agar, 0,5 g leucine, 30.24 g Pipes and 12 g of a solution of 50% (w/w) NaOH to raise the pH to dissolve Pipes (pH 6.8). The dye solution was finally poured along the glass wall. Colony diameters of two colonies were measured using three biological and two technical replicates.

For the germination assay, sporangiospores were inoculated in RPMI-1640 and an FBS containing liquid medium (20 g glucose, 10 g peptone and 100 ml heat inactivated FBS [Biosera, Kansas City, MO, USA] per liter); the final concentration of spores was 10^6^/mL. RPMI-1640 liquid medium was supplemented with 10 µM of FeCl_3_, FeSO_4_, FeH_8_N_2_O_8_S_2_, Deferoxamine, ferrioxamine B, Deferasirox or Deferasirox-Fe(III) complex, enterobactine, and ferrienterobactine. The inoculated media were stored at 4°C for 16 hours. Next day, the germinated spores were counted at 3.5, 4, 5 and 6 hours postinoculation. The experiment was performed using three biological and two technical replicates and more than 1000 spores were analyzed and counted per sample.

### Molecular techniques and sequence analysis 

Genomic DNA and RNA samples were purified from the mycelia using the ZR Fungal/Bacterial DNA MiniPrep (Zymo Research) and the Quick-RNA MiniPrep kit (Zymo Research), respectively, according to the manufacturers’ instructions. Genes were amplified by PCR using the Phusion Flash High-Fidelity PCR Master Mix (Thermo Fischer Scientific, Waltham, MA, USA) and the primers presented in Supplementary Table S2. Primer and oligonucleotide sequences were designed using the *M. lusitanicus* CBS277.49v2.0 genome database (DoE Joint Genome Institute; https://mycocosm.jgi.doe.gov/Mucci3/Mucci3.home.html) 69. Sequencing was commercially performed by the LGC Genomics (Berlin, Germany).

### *In silico* analysis and homology modelling

In order to identify siderophore transporters, we retrieved all available annotated protein sequences from the NCBI database (https://www.ncbi.nlm.nih.gov/) 70 using the keywords "siderophore transporter" and "siderochrome transporter". Hits were filtered to include only fungal sequences. The initial dataset contained a total of 6,997 sequences. Partial sequences and those associated with siderophore biosynthesis were excluded while redundant identical sequences were collapsed by using SeqKit v2.1.1 software 71. The filtered sequences were aligned with MAFFT v7.453 72 employing the FFT-NS-i iterative refinement algorithm. From this alignment, we manually extracted sequences that showed substantially divergent patterns compared to the majority of the dataset. These outlier sequences were subjected to BLAST analysis against the TCDB database (https://www.tcdb.org) 73 for further identification. Sequences that were not confirmed as siderophore transporters were subsequently removed from the dataset. The refined dataset comprised 3,298 sequences. A multiple sequence alignment was generated using the G-INS-i algorithm of the MAFFT software, and a hidden Markov model (HMM) profile was built with HMMER v3.3.2 74 package (available as supplementary file). This HMM profile was subsequently used to refine the results of NCBI blastp searches. Prior to the final analysis sequences were aligned using MAFFT with the FFT-NS-i algorithm, and a maximum likelihood tree was generated with FastTree v2.1.10 75 with default settings. To reduce the dataset, closely related sequences were excluded by using Treemmer v0.3 76 with an RTL threshold of 0.9. For the final phylogenetic analysis of the identified siderophore transporters, sequences were aligned using the G-INS-i algorithm of MAFFT. The best-fit evolutionary model for the dataset was selected using the inbuilt model selection 77 function of IQ-TREE v2.0.7 78. Maximum likelihood phylogenetic inference was performed using IQ-TREE with statistical support evaluated using the ultrafast bootstrap 79 method with 5,000 replicates.

Transmembrane domains of the Sit1 protein were predicted using the online tool HMMTop (http://www.enzim.hu/hmmtop 80.

Homology models of *M. lusitanicus* Sit1 and Ramachandra plot were generated with SWISS-MODEL using its amino acid sequence as template (MUCCIDRAFT_154647).

### Quantitative reverse transcription PCR (RT-qPCR) analysis 

Reverse transcription was carried out with the Maxima H Minus First Strand cDNA Synthesis Kit (Thermo Fischer Scientific, Waltham, MA, USA) using random hexamer and oligo (dT)18 primers, following the instructions of the manufacturer. The RT-qPCR experiments were performed in a CFX96 real-time PCR detection system (Bio-Rad) using the Maxima SYBR Green qPCR Master Mix (Thermo Fischer Scientific, Waltham, MA, USA) and the primers presented in Supplementary Table S2. The relative quantification of the gene expression was achieved with the 2^-ΔΔCt^ method 81 using the actin gene of *M. lusitanicus* as a reference 82. Experiments were performed in biological and technical triplicates.

### Knockout of the *sit1* and *fet4a* genes by the CRISPR-Cas9 method 

The protospacer sequences designed to target the DNA cleavage in *sit1* (protein ID: 1388546) and *fet4a *(protein ID: 1364625) were 5′-ATGGTAGGATCAATGACAGT- 3′ and 5′-AGAGTTTGTAGAACG TAATG- 3′, respectively. Using these sequences, the Alt-R CRISPR crRNA and Alt-RCRISPR-Cas9 tracrRNA molecules were designed and purchased from Integrated DNA Technologies (IDT, Coralville, IA, USA). To form the crRNA:tracrRNA duplexes (i.e. the gRNAs), the Nuclease-Free Duplex Buffer (IDT) was used according to the instructions of the manufacturer. Genome editing strategy and homology driven repair (HDR) followed the set-up described earlier 83. Deletion cassette functioning also as the template DNA for the HDR was constructed by PCR using the Phusion Flash High-Fidelity PCR Master Mix (Thermo Fischer Scientific, Waltham, MA, USA). Two fragments, upstream from the start codon and downstream from the stop codon of the targeted gene and the *M. lusitanicus* *pyrG *gene (CBS277.49v3.0 genome database Protein Id: 1355982) or* M. lusitanicus* *leuA* gene (CBS277.49v3.0 genome database Protein Id: 1545615) along with its own promoter and terminator sequences were amplified using gene specific primer pairs (see Supplementary Table S2). The amplified fragments were fused in a subsequent PCR using nested primers (see Supplementary Table S2); the ratio of the fragments in the reaction was 1:1:1 (Supplementary Figure S3).

To introduce the elements of the CRISPR-Cas9-mediated gene knockout (i.e., the gRNA, the Cas9 enzyme and the template DNA) into the fungus, the PEG-mediated protoplast transformation method was used as described earlier 60. For the gene knockout, 5 μg template DNA (i.e., the disruption cassette), 10 μM gRNA and 10 μM Cas9 nuclease were added to the protoplasts in one transformation reaction. In each case, transformants were selected on solid YNB medium by the complementation of the uracil and/or leucine auxotrophy of the MS12 strain. From each primary transformant, monosporangial colonies were formed under selective conditions.

Single knockout of the *sit1* and the *fet4a *genes resulted in the *sit1*Δ and the *fet4a*Δ strains, respectively. Simultaneous knockout of the two genes led to the construction of the *sit1*Δ:*fet4a*Δ strain. Knockout of the genes was demonstrated by PCR analysis of the mutant strains amplifying the expected fragments in each case (Supplementary Figure S4 and Figure S5). From all transformation experiments, two transformants were finally selected and homologous replacement was confirmed by PCR (Supplementary Figure S4 and Figure S5) and Southern blotting (Figure 3). In brief, primers; P1 and P2 (Supplementary Table S2), were used to identify the integration of the *pyrG* or *leuA* gene at the *sit1* or *fet4a* loci. The genomic DNA of the deletion candidates and MS12 strain were digested with *EcoR*I or *EcoR*V and Southern blotting was performed with the 5′ fragment as a probe as described 84.

Mutants proved to be mitotically stable retaining the integrated fragment even after 20 cultivation cycles. In all further analyses, two independently derived isolates were tested for each mutant strain.

### Determination of ferric reducing capacity

The assay was performed using ferric reducing antioxidant power (FRAP) reagent contained 4 mL of 10 mmol/L TPTZ diluted in 40 mmol/L hydrochloric acid, 4 mL of 20 mmol/L iron(III) chloride solution, 20 mL of sodium acetate buffer (300 mmol/L, pH 3.6) and 2.2 mL of distilled water. The TPTZ solution was heated to 50°C in a water bath before addition to FRAP reagent, and the prepared reagent was incubated at 37°C until the measurement. For the reaction 10^6^ spores/mL of the fungal strains were pre-cultivated in 2 mL liquid Czapek-Dox medium without iron for one day, then 200 μL of FRAP reagent was added. The reaction was then incubated at 37°C for 30 min, and the absorbance was measured at 593 nm using a SPECTROstar Nano microplate reader (BMG Labtech, Offenburg, Germany). The experiment was performed using three biological and two technical replicates.

### Quantification of Deferoxamine in liquid media

For deferoxamine quantification, the fungus was pre-cultivated for one day in iron-free liquid Czapek-Dox medium using 10^6^ spores/mL. Following this pre-incubation, 10 µM of ferrioxamine B was added to the medium. After an additional 24-hour incubation, the fungal biomass was removed by centrifugation and filtration through a 0.45 µm membrane filter. LC-HRMS measurements were performed using a DionexUltimate 3000 UHPLC system (Dionex) coupled to an Q Exactive Plus hybrid quadrupole-Orbitrap mass spectrometer that was equipped with a heated electrospray ionization (HESI-II) source. Siderophores were separated using a Gemini-NX C18 (3 μm, 150 x 2 mm) column. The mobile phases consisted of water (A) and acetonitrile/methanol (1/1) (B) both supplemented with 0.1% formic acid. A gradient elution profile was applied as follows: 0-2 min isocratic with 5% B; 2-13 min from 5 to 95% B; 13-19 min isocratic with 95% B; 19-19.5 min from 95 to 5%B; 19.5-24 min isocratic with 5% B. The flow rate was 0.2 mL/min, and the injection volume was 3 µL. The column temperature was maintained at 25°C. The ion source had the following settings: probe heater temperature 300°C, ion transfer capillary temperature 320°C, spray voltage 3.5 kV, sheath gas flow rate 30 arbitrary unit, auxiliary gas flow rate 10 arbitrary unit and S-lens RF level 50 arbitrary unit. Siderophores were detected with parallel reaction monitoring (PRM) method using the protonated molecular ions [M+H] + of ferroxamine B at 614.266 m/z and deferroxamine B at 561.359 m/z. Precursors were selected in the quadrupole with an isolation window of 0.4 m/z and fragmented with HCD using 30% collision energy. MS2 spectra were recorded at 17,500 resolution with a minimum automatic gain control target of 5.00×105 and a maximum injection time of 64 ms. LC-HRMS data were acquired and processed using Trace Finder 4.0 software. The standard curve was established using 10 µmol/mL of deferoxamine solution in the concentration range of 1-10 µmol/mL. The experiment was performed using two biological and six technical replicates.

### Susceptibility tests

Sensitivity of the fungal strains to different antifungal agents was examined in a 96-well microtiter plate assay. The susceptibility test was performed according to the CLSI recommendation in three biological replicates. Ketoconazole (Alfa Aesar, Haverhill, MA, USA), itraconazole (Across Organics, Waltham, MA, USA), fluconazole (Alfa Aesar, Haverhill, MA, USA),), ravuconazole (Sigma Aldrich, St. Louis, MO, USA), posaconazole (Sigma Aldrich, St. Louis, MO, USA), isavuconazole (Sigma Aldrich, St. Louis, MO, USA), and amphotericin B (Sigma-Aldrich) were dissolved in dimethyl sulfoxide to prepare the stock. These stocks were then diluted with liquid RPMI-1640 medium. Final concentrations of azoles and AmB in the wells ranged from 0.125 to 16 μg/mL. Inocula were prepared and diluted in liquid RPMI-1640. Plates were incubated for 48 h at 25°C. For the antifungal susceptibility test, *Candida krusei* ATCC 6258 was used as a reference strain. The experiment was performed using three biological and two technical replicates.

### Survival assay in *Galleria mellonella* larvae 

Spores were resuspended in insect physiological saline (IPS, 50 mM NaCl, 5 mM KCl, 10 mM EDTA and 30 mM sodium citrate in 0.1 M Tris-HCl, pH 6.9) 85. *G. mellonella* larvae (BioSystems Technology, TruLarv) were inoculated with 10^5^ fungal cells in 20 µL IPS via the last proleg using 29-gauge insulin needles (BD Micro-Fine). For each *M. lusitanicus *strain, 20 larvae were infected. For IPS-treated (uninfected) and witness control (no injections, uninfected), 20 animals were utilized too. The larvae were maintained at 28°C, and their survival was monitored daily for 6 days. The results shown are representatives of at least three independent experiments using two biological and three technical replicates.

### Statistical analysis 

All measurements were performed in at least two technical and three biological replicates. Statistical significance was analyzed by t-tests, One-way or Two-way ANOVA followed by Dunnett’s multiple comparisons test using Microsoft Excel of the Microsoft Office package or GraphPad Prism 7.00 (GraphPad Software, La Jolla, California USA) as appropriate. P values less than 0.05 were considered statistically significant. In *in vivo* survival experiments, differences between the pathogenicity of the fungal strains were compared by the Gehan-Breslow-Wilcoxon test and Mantel-Cox test values less than 0.05 were considered statistically significant.

## CONFLICT OF INTEREST

The authors declare that they have no conflict of interests.

## SUPPLEMENTAL MATERIAL

Click here for supplemental data file.

Click here for supplemental data file.

Click here for supplemental data file.

All supplemental data for this article are available online at www.microbialcell.com/researcharticles/2025a-vago-microbial-cell/.
