# Supplementary material for: Role of the putative sit1 gene in normal germination of spores and virulence of the Mucor lusitanicus
Source: Microb Cell. 2025 Aug 12;12:195–209. doi: 10.15698/mic2025.08.856 (PMC12380104; doi:10.15698/mic2025.08.856)
Supplement: Supplementary file 1 [file mic-12-195-s01.pdf]

Supplemental materials

Role of the putative *sit1* gene in normal germination of spores and virulence of the *Mucor lusitanicus*

Bernadett Vágó[1,2], Kitti Bauer[1,2], Naomi Varghese[1,2], Sándor Kiss-Vetráb[1,2], Sándor Kocsubé[1,2], Mónika Varga[1,2], András Szekeres[1,2], Csaba Vágvölgyi[1,2], Tamás Papp[1,2,3]¶, Gábor Nagy[1,2,3]*¶

[1]Department of Biotechnology and Microbiology, University of Szeged, Szeged, Közép fasor 52, Hungary

[2] HUN-REN-SZTE Fungal Pathomechanisms Research Group, University of Szeged, Szeged, Hungary, Szeged, Közép fasor 52, Hungary

[3]University of Szeged, Centre of Excellence for Interdisciplinary Research, Development and Innovation (SZTE IKIKK), Fungal Pathomechanisms Research Group, Szeged, Hungary, Szeged, Közép fasor 52, Hungary

*Corresponding author to: Department of Microbiology, University of Szeged, Szeged, Közép fasor 52, Hungary

E-mail address: nagy.gabor.04@szte.hu (G.N.)

These authors contributed equally to this work

Running title: Siderophore transporter 1 of *Mucor lusitanicus*

Keywords: mucormycosis, siderophore, Sit1/ARN3, deferoxamine, iron acquisition

Figure S1 Maximum likelihood phylogram generated with IQ-TREE using the LG+R6 evolutionary model. Sequences annotated as siderophore transporters are highlighted in bold. The siderophore transporter examined in this study is highlighted in red. Sequences of proteins AZR1 and SGE1 are used as outgroup during the phylogenetic reconstruction. Staistical support of the best tree was calculated with Ultrafast Bootstrap method in 5000 replicates. Only values higher than 95 percent are shown on branches.

A

```
         10        20        30        40        50        60
....|....|....|....|....|....|....|....|....|....|....|....|
MKFRLPSIFPAAAIEETRDNKEPVDAELSNDDEKRVAQDNSSVMNTKEEIPTQESDAASE
         70        80        90       100       110       120
....|....|....|....|....|....|....|....|....|....|....|....|
RDSTAEFQYGVKSVQASLQVWTKSHLIWAYGLIWTISFVLALSSGINSTLTPYVTSNFNE
        130       140       150       160       170       180
....|....|....|....|....|....|....|....|....|....|....|....|
HSLTATTQVISSMISGLIVLPYAKFINIFGRPLGFALMVGSMTVGLIMMAACQNVTTYCA
        190       200       210       220       230       240
....|....|....|....|....|....|....|....|....|....|....|....|
AQVFYSVGSSTINFSMTIFIADTTSLKNRAFMIAFTASPWIATVWAYGPAAQHILDTIGF
        250       260       270       280       290       300
....|....|....|....|....|....|....|....|....|....|....|....|
RWNFGIWAIVIPIVCAPLCGLFYYNYLKAVKAGLIVKPKSNRTFAQSCYYYAVEFDVIGL
        310       320       330       340       350       360
....|....|....|....|....|....|....|....|....|....|....|....|
LLICAGLALFLLSFNLYSYQPGEWSSPLIVCFLVFGVLLMIAFGCWEKWGAPITFIPWSL
        370       380       390       400       410       420
....|....|....|....|....|....|....|....|....|....|....|....|
LMDRTVFFTYTMAVSLYLAWYLWDSFFYSYLIVVYNLDISQATYITNIYTIGATFWSLVM
        430       440       450       460       470       480
....|....|....|....|....|....|....|....|....|....|....|....|
GLVIMYNGRLKWQAFYFGVPITMLGVGLMIMFRQPDVNIGYIVMCQIFIAFGGGTLVICE
        490       500       510       520       530       540
....|....|....|....|....|....|....|....|....|....|....|....|
QMTVMAVSSHQFVPAVLSMENMVISIGSAVGSAIAGAMWTGIFPQKLAKYLPASALGDLQ
        550       560       570       580       590       600
....|....|....|....|....|....|....|....|....|....|....|....|
SIYGSITVQSSYAIGSPERIGINLAYSETQRYMLIAATCLYVITWASVAMWKDVDVRQIK

....|..
KTGLRQL
```

B

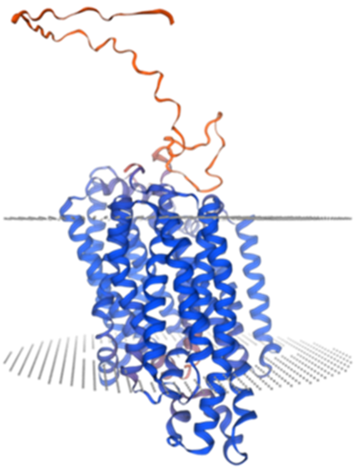

Figure S2 Putative amino acid sequence of the Sit1 protein. Black letters indicate amino acid sequence motifs at the inner part of the membrane, red letters indicate the trans membrane helixes, while white letter indicate the amino acid sequence motifs at the outer part of the membrane (A), and. predicted tertiary structures of the proteins Sit1 of *Mucor lusitanicus*. The dotted line represents the membrane (B).

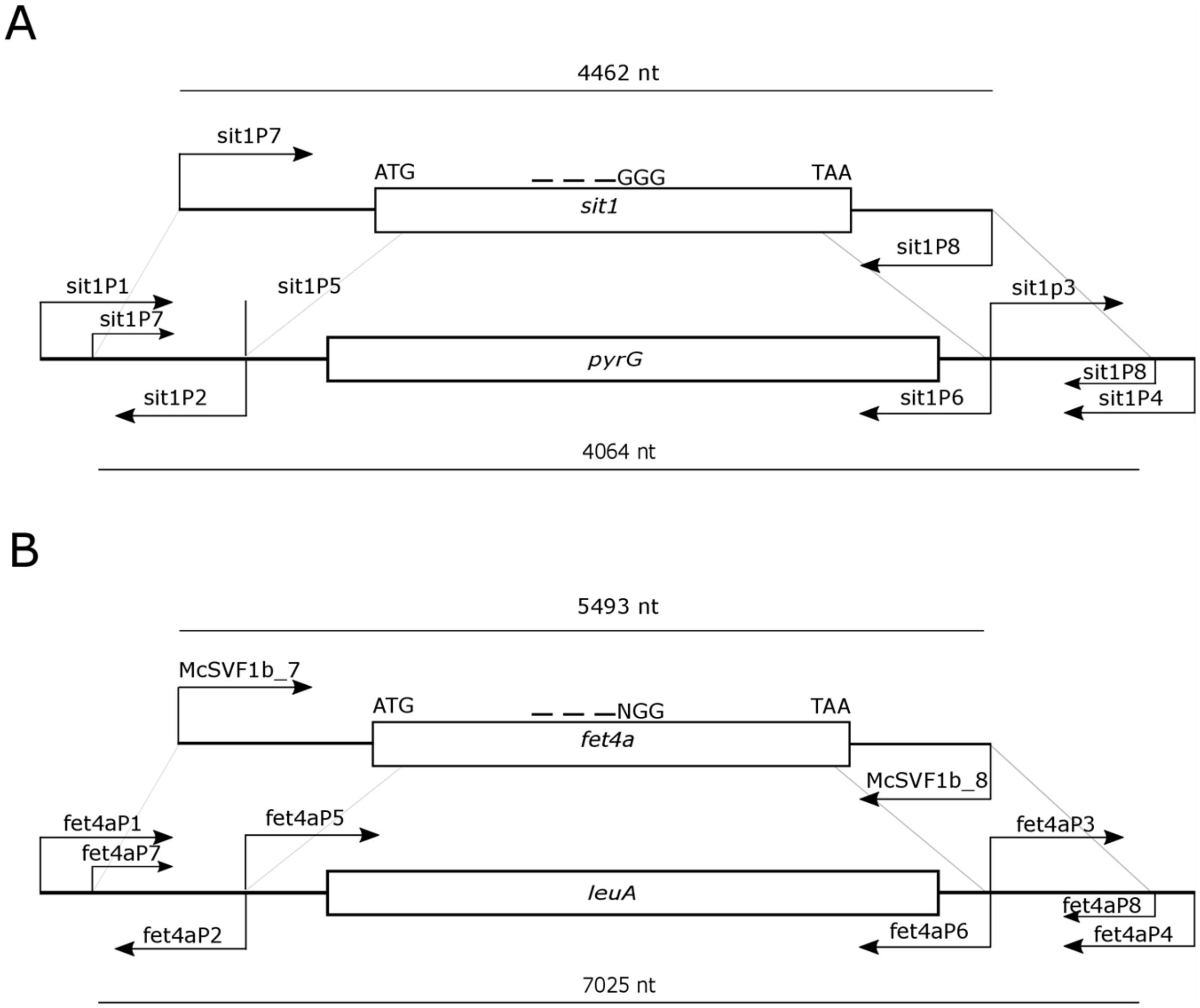

Figure S3 Genome editing strategy designed to disrupt the *sit1* and *fet4a* genes of Mucor lusitanicus using the CRISPR-Cas9 method. HDR was performed using the deletion cassette/template DNA containing either the *pyrG* (A) or the *leuA* (B) gene as selection markers. Positions of the primers used to analyze or amplify the constructs are presented (for the nucleic acid sequences of the primers, see Supplementary Table S1). NGG indicates the PAM sequence while the arrows show the orientations of the primers.

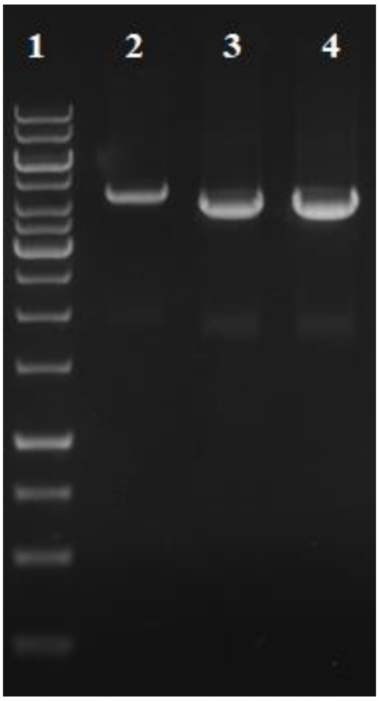

Figure S4 PCR analysis of the transformants. (1) GeneRuler 1 kb DNA ruler (Thermo Scientific); (2) MS12+*pyrG*; (3) *sit1*Δ/1; (4) *sit1*Δ/2. For the primer sequences and the primers used in the PCR experiments, see Supplementary Table S2.

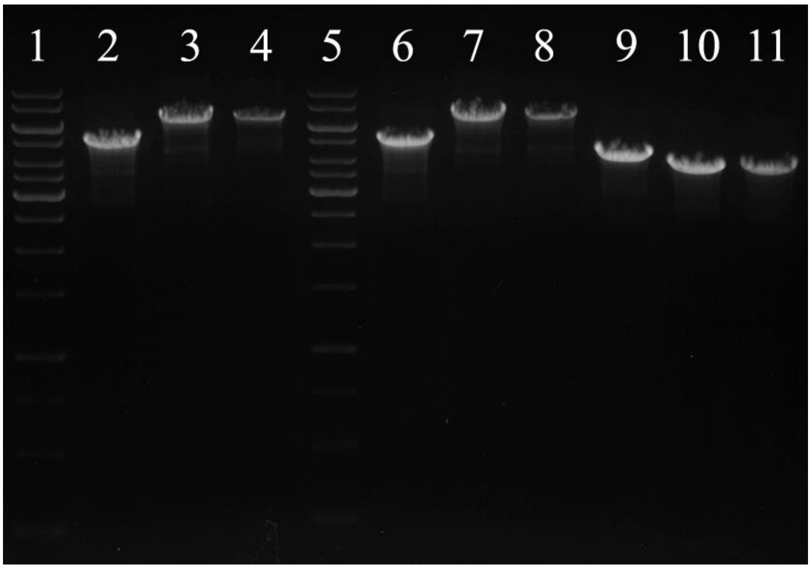

Figure S5 PCR analysis of the transformants. (1) GeneRuler 1 kb DNA ruler (Thermo Scientific); (2) MS12+*pyrG*; (3) *fet4a*Δ/1; (4) *fet4a*Δ/2; (5) GeneRuler 1 kb DNA ruler (Thermo Scientific); (6) MS12+*pyrG* (7) *sit1*Δ:*fet4a*Δ/1; (8) *sit1*Δ:*fet4a*Δ/2; (9) MS12+pyrG (10) *sit1*Δ:*fet4a*Δ/1; (11) *sit1*Δ:*fet4a*Δ/2. For the primer sequences and the primers used in the PCR experiments, see Supplementary Table S1.

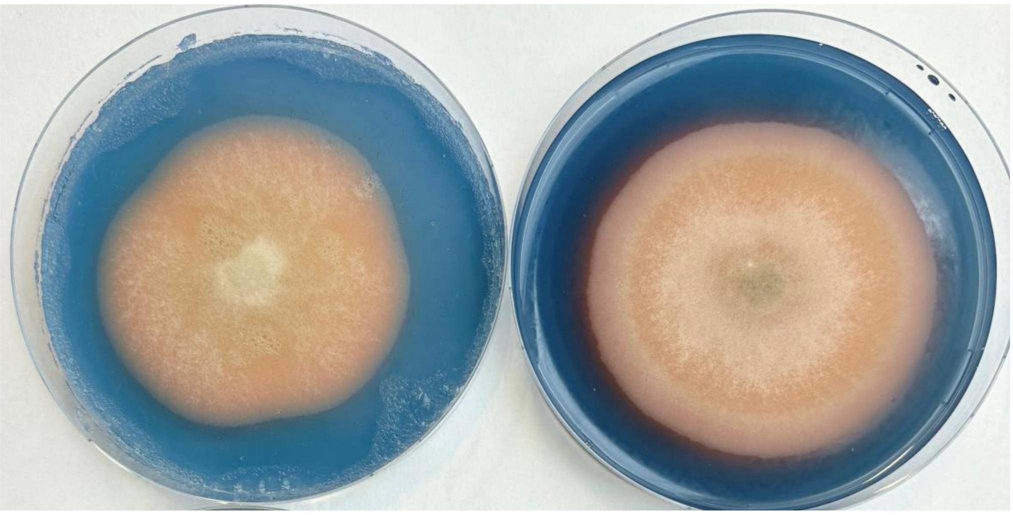

Figure S6 Siderophore production of *sit1*Δ (left) and MS12+*pyrG* (right) strains on CAS agar after six days of incubation. 10^6 spores/ml were inoculated to each plate.

TABLE S1 Sequences used for the evolutionary model

TABLE S2 Primers used in the present study

Primers	Sequence 5’- 3’	Amplified DNA
	Phusion PCR for *sit1* deletion	
sit1P1	ATTACAATCAACCCTATCGCTG	promoter of *sit1*
sit1P2	TAGGTGCATACAGGTAGGAC	
sit1P3	ATGCGTAAATGTAATCTCCCGT	terminator of *sit1*
sit1P4	TGTATGGTGAAGAGTGGTGATAGG	
sit1P5	TGAATATTCAAATGGGTCCTACCTG<br>TATGCACCTATGCCTCAGCATTGGTACTTG	*pyrG* with own promoter and terminator
sit1P6	GTGACAGGCTTAAACGGGAGATTAC<br>ATTTACGCATGTACACTGGCCATGCTATCG	
sit1P7	GTCTTTCAAATCAAACCAGATACGG	final PCR product for transformation
sit1P8	ACGATCCACTACAAACACTAGCA	
	Phusion PCR for *fet4a* deletion	
fet4aP1	ATGATGTGGTAATTCGTGTGCT	promoter of *fet4a*
fet4aP2	TGTGGAGCCAAATGTCATTGT	
fet4aP3	TAACTTTCTGACGCCTTTGCT	terminator of *fet4a*
fet4aP4	ATGATACACTAAACCCACTCGT	
fet4aP5	TCTCTCTCTCTAAAACAATGACATTTGGCT<br>CCACACTGCAGGATGGGACAAGGTATATAT	*pyrG* with own promoter and terminator
fet4aP6	TCCAGAAAAAGTGAAGCAAAGGCGTCAGAA<br>AGTTACTGCAGTAGCTGTTGATGTTGTTGT	

fet4aP7<br>ffet4aP8	GGCTATACTTCTCGCTCAATTATGG<br>GTAGTAGTCATATCCACATCATCGG	final PCR product for transformation
Primers for qRT-PCR experiments		
sit1rtfw<br>sit1rtrev	TTTCGGTATTTGGGCTATTGTC<br>AATGTTCTGTTGCTCTTGGG	*sit1*
MCactinF<br>MCactinR	CACTCCTTCACTACCACCGCTGA<br>GAGAGCAGAGGATTGAGCAGCAG	actin
Ml75421RTfw<br>Ml75421RTrev	CTGATGACAATGAGCCTAATCC<br>ACCAGTACCAATAACATCACCA	low affinity iron permease
Ml149242RTfw<br>Ml149242RTrev	CTATTCACCCTCATCCTGTTACTC<br>CTCCATTCCATAACATCGCCA	low affinity iron permease
Ml153842RTfw<br>Ml153842RTrev	GGCGATACCATTTCTTACAGAG<br>CCGACAACAAATAACCAGTAGAG	*ftr1*
Ml156579RTfw<br>Ml156579RTrev	TACTATCGGCACCATTGTCAG<br>CTTCTTCGCCCACAATAGCA	*ftr1*
Ml187130RTfw<br>Ml187130RTrev	GACTCCAATAGCCATCCTTTCC<br>AATCCCTCAGCATCTACACGA	copper dependent iron oxidases
Ml156933RTfw<br>Ml156933RTrev	GACTCCAATAGCCATCCTTTCC<br>AATCCCTCAGCATCTACACGA	copper dependent iron oxidases
Ml142925RTfw<br>Ml142925RTrev	TACATTACTGCTCTCATCACACTG<br>ATCTCCACCTCATCTCCCTG	copper dependent iron oxidases
77308fw<br>77308rev	TGCTATCAGTTGAACAGAATCC<br>TCTTGATTGAAACCATCTCGG	ferric reductase
115816fw<br>115816rev	CTCATACACTCCAGCACCAG<br>GAGATACCTGTCAACAGCGA	ferric reductase
114066fw<br>114066rev	TTTCCATCAGAACCACTACCA<br>CGAACCACATAATCAAGACGG	ferric reductase
85232fw<br>85232rev	CTAACCTCTTACCTTGCCCTTGTC<br>GATAGCAGCGTCGTATTGTCTC	ferric reductase

TABLE S3 Features of strains used in this study

Strain	Protein ID(s)	Gene(s)	Genotype	Description
MS12			*leuA*$^-$;*pyrG*$^-$	Parental strain, leucin and uracil auxotrophic
MS12+*pyrG*	1355982	*pyrG*	*leuA*$^-$;*pyrG*$^+$	Uracil auxotrophy was complemented, leucin auxotrophic
*sit1*Δ	1388546	*sit1*	*leuA*$^-$;*pyrG*$^+$	Deletion of *sit1,* leucin auxotrophic
*fet4a*Δ	1364625	*fet4a*	*leuA*$^+$;*pyrG*$^-$	Deletion of fet4a*,* uracil auxotrophic
*sit1*Δ*:fet4a*Δ	1388546:1364625	*sit1*:*fet4a*	*leuA*$^+$;*pyrG*$^+$	Deletion of *sit1* and *fet4a*, prototrophic

Table S4 MICs of the azoles and AmB against the *sit1*Δ, *fet4a*Δ and *sit1*Δ*:fet4a*Δ mutants

	MIC (µg/ml)					
Strain	Ketoconazole	Itraconazole	Fluconazole	Posaconazole	Izavuconazole	Amphotericin B
MS12+*pyrG*	16<	16<	16<	8	16<	1
*sit1*Δ	16<	16<	16<	4 (MIC50)	16 (MIC50)	0.5 (MIC50)
*fet4a*Δ	16<	16<	16<	8<	16<	1
*sit1*Δ:*fet4a*Δ	16<	2	16<	1	8	1
