# Supplementary figures and images for: Role of the putative sit1 gene in normal germination of spores and virulence of the Mucor lusitanicus
Source: Microb Cell. 2025 Aug 12;12:195–209. doi: 10.15698/mic2025.08.856 (PMC12380104; doi:10.15698/mic2025.08.856)

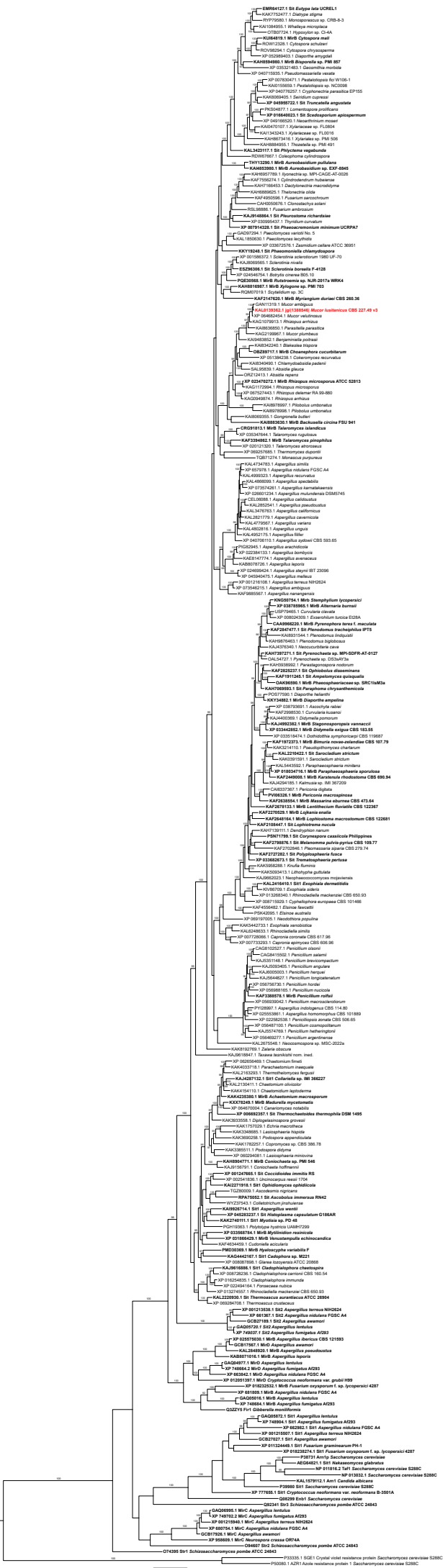

Supplement: Supplementary file 2 [file mic-12-195-s02.pdf]
